# Editorial: Functional heterogeneity of stem cells

**DOI:** 10.3389/fcell.2023.1179911

**Published:** 2023-03-17

**Authors:** Pyotr A. Tyurin-Kuzmin, Yohei Hayashi, Konstantin Kulebyakin

**Affiliations:** ^1^ Department of Biochemistry and Regenerative Biomedicine, Faculty of Medicine, Moscow State University, Moscow, Russia; ^2^ iPS Cell Characterization and Development Team, RIKEN BioResource Research Center, Tsukuba, Ibaraki, Japan; ^3^ Institute for Regenerative Medicine, Moscow State University, Moscow, Russia

**Keywords:** stem cells, heterogeneity, miRNA, senescence, regenerative medicine

Regulation of the functional activity of stem cells is extremely important for their coordinated action in the processes of tissue repair and regeneration, as well as maintenance of its homeostasis. We now understand that stem cells are not just progenitors but are capable of regulating their host tissue’s function. This includes regulation of proliferation, migration, and differentiation of other cells, as well as secretion of paracrine factors, extracellular vesicles, and regulatory microRNAs. A wide variety of mechanisms and forms of regulation of stem cells hormonal sensitivity ensures harmonious interaction between the body, tissue, and stem cells, finely adjusting their functioning to dynamically changing conditions. This diversity is based on the functional heterogeneity of stem cell populations in the human body.

The functional heterogeneity of stem cells manifests itself at several levels. First, stem cells differ in their properties when they are isolated from different individuals, even from the same line of animals. This is especially noticeable and important in practical terms when it comes to the use of primary cultures of human stem cells. Secondly, stem cells of even the same type differ when they are isolated from different organs. For example, multipotent mesenchymal stromal cells (MSCs) of adipose tissue differ in their proliferative, and differentiation potential, as well as in their immunomodulatory ability when isolated from various adipose tissue depots (Kulebyakin et al., 2022). Further, stem cells are heterogeneous in their sensitivity to regulatory hormones. Thus, it was shown that individual MSCs are sensitive to various hormones that activate GPCR (Kotova et al., 2014). At the same time, cells show plasticity, signal heterogeneity reappears in clones of a single cell (Tyurin-Kuzmin et al., 2020).

As a result, at the level of the whole organism, functional heterogeneity allows us to achieve two goals: to ensure that stem cells are in different states (for example, part of the stem cells is in an undifferentiated state as a tissue regenerative potential), and on the other hand, to provide sensitivity to a wide range of regulatory influences at the population level.

This Research Topic has invited original research articles and reviews focused on all aspects of functional heterogeneity of stem cells including but not limited to the links between the phenotype of stem cells and their functions, functional analysis of subpopulations of stem cells, regulation of stem cell heterogeneity by signaling molecules and microRNAs, and influence of cellular senescence and metabolic disorders on the sub-population composition of stem cells. The Research Topic collected three original research articles.

The functional heterogeneity of stem cells is a big enough problem for the use of these cells in regenerative medicine since they can lead to unpredictable side effects of cell therapy. A possible solution to the problem of primary stem cell cultures is the use of induced pluripotent cells (iPSCs). Although their cultures also show cell-to-cell heterogeneity (Hayashi et al., 2019), the results are more reproducible. Akaba et al. showed the contribution of microRNA-514a (miR-514a) to the regulation of the differentiation potential of iPSCs in the neuronal direction. Increased expression of this siRNA leads to a significant increase in the efficiency and homogeneity of iPSC differentiation towards neurons. The authors also analyzed polymorphisms in the sequence encoding this miRNA among primates. As shown, several polymorphisms lead to a decrease in the level of expression of this miRNA in stem cells of the nervous tissue, which may be associated with features of brain development. Their results provide new insights into the roles of miRNAs in the regulation of robust development of nervous tissues using iPSC-derived neurons.

Another significant challenge faced by specialists in the field of regenerative medicine is the heterogeneity of stem cells associated with cellular senescence and aging. Voynova et al. showed that extracellular vesicles produced by senescent multipotent mesenchymal stromal cells (MSCs) induce insulin resistance in non-senescent cells. These extracellular vesicles contain miRNAs that specifically and persistently up-regulate insulin-associated intracellular signaling cascades, which in turn leads to a reduced response to insulin action. These findings suggest that cells used for secretome production for further medical use should be closely monitored and assessed for accumulation of senescent phenotype.

Immune responses pose a significant problem when stem cells are considered for cell-based therapy. In their work, Karpenko et al. addressed the interplay between the immune system and MSCs. Despite having an immunogenic marker, MSCs are able to create fully functional ectopic hematopoietic foci under renal capsule. The key role in this process is played by a specific Nestin-positive subpopulation of MSC, which are immunoprivileged. That opens a number of opportunities regarding modulation of immune response to cell therapy as well as understanding of mechanisms of formation of immunoprivileged compartments within the organism.

Studies of stem cell heterogeneity is an actively growing field in modern biomedical science. It becomes more and more clear that many functions and properties attributed to stem cells are realized not through individual cells but on the level of the whole population. For example, a widely known ability of stem cells to respond to a big variety of hormonal stimuli is not because of the array of receptors presented on each cell but because of multiple subpopulations each sensitive to individual hormone. This means that functional heterogeneity of stem cell population is not a coincidence, but an important feature that defines the ability of stem cells to perform their functions. Understanding this calls for a change of a modern cytological paradigm suggesting the equality of individual stem cells in population.
